# Differential expression profiles and bioinformatics analysis of microRNAs in brown adipose tissue dysfunction induced by chronic intermittent hypoxia in obstructive sleep apnea

**DOI:** 10.3389/fcell.2025.1598018

**Published:** 2025-08-15

**Authors:** Meilin Ji, Yaopeng Guo, Jinjie Zhang, Shu Lin, Liangyi Li, Qingshi Chen

**Affiliations:** ^1^ The Second Clinical Medical College, Fujian Medical University, Quanzhou, China; ^2^ The Second Affiliated Hospital of Fujian Medical University, Quanzhou, China; ^3^ Department of Endocrinology and Metabolism, The Second Affiliated Hospital of Fujian Medical University, Quanzhou, China

**Keywords:** obstructive sleep apnea, brown adipose tissue, microRNA, metabolic syndrome, bioinformatic analysis

## Abstract

Obstructive sleep apnea (OSA) is a sleep-related respiratory disorder. Although recent studies have shown that OSA may be an alterable risk factor for metabolic syndrome (MS), the precise mechanism remains unknown. This study was designed with the purpose of identifying differentially expressed microRNAs (DEmiRs) in OSA-induced brown adipose tissue (BAT) injury. In this study, mouse models of chronic intermittent hypoxia (CIH)-related BAT injury were established using APOE mice. The microRNAs (miRNAs) expression profiles of the CIH-caused BAT injury were analyzed by the miRNA sequencing technology. The miRNA-seq data were analyzed using Gene Ontology (GO) analysis and Kyoto Encyclopedia of Genes and Genomes (KEGG) analysis. An analysis of real-time quantitative PCR (RT-qPCR) confirmed the presence of several typical miRNAs. Ultimately, we constructed a network to illustrate the correlation between the miRNAs and target genes. In the CIH-induced BAT damage mouse models, 7 miRNAs experienced an upregulation, and 16 miRNAs underwent a downregulation. Six DEmiRs were confirmed using RT-qPCR. Additionally, GO and KEGG analyses were adopted to annotate the potential biological role of miRNAs. As a final step, we construct a miRNA–mRNA network for predicting miRNAs target genes. In conclusion, we first discovered that OSA-induced BAT dysfunction is associated with abnormal miRNA expression. This study exhibited a novel understanding of the potential molecular mechanism of OSA-related MS.

## Introduction

As a highly prevalent disorder, obstructive sleep apnea (OSA) is defined by the repeated narrowing or closure of the airway during sleep. The airway collapse leads to increased chronic intermittent hypoxia (CIH), sleep fragmentation, and negative intrathoracic pressure. Considerable evidence has been proved to support an independent correlation of OSA with comorbidities including metabolic dysfunction (Almendros et al., 2022), atherosclerotic disease (Sapiña-Beltrán et al., 2022), and pulmonary hypertension (Sharma et al., 2021). Multiple experimental studies have proved that OSA is closely correlated with metabolic syndrome (MS) (Cui et al., 2018; Dahan et al., 2022; Huang et al., 2022). While these clinical links are well-documented, the search for biological mediators has identified brown adipose tissue (BAT)-a key thermogenic organ involved in energy expenditure-as a potential target for OSA-related metabolic dysfunction. Brown adipocytes are considered an effective target for treating OSA-related metabolic disorders (Dong et al., 2018). Brown adipocytes uniquely express uncoupling protein 1 (UCP1), enabling nonshivering thermogenesis through mitochondrial uncoupling. This process consumes glucose and lipids, making BAT a critical regulator of systemic metabolism. Dysfunctional BAT is implicated in insulin resistance and dyslipidemia---hallmarks of metabolic syndrome (Dong et al., 2018; Saito and Okamatsu-Ogura, 2023). Thus, elucidating BAT pathology in OSA may reveal actionable therapeutic targets. Despite this potential, the molecular mechanisms linking OSA-induced hypoxia to BAT dysfunction remain poorly characterized. In particular, the role of epigenomic regulators like microRNAs (miRNAs) in this context is entirely unexplored.

miRNAs are small non-coding RNAs that fine-tune gene expression by targeting mRNAs for degradation or translational repression. Critically, miRNAs such as miR-669a-5p directly regulate UCP1 expression and adipocyte browning (Tan et al., 2022). Hypoxia-responsive miRNAs (e.g., miR-210) further modulate metabolic adaptation in adipose tissue (Caca et al., 2025). The importance of miRNAs has been noted in prior studies, which get involved in innumerable biological processes, such as cell proliferation, differentiation, apoptosis, and tumorigenesis (Naqvi et al., 2022; Tealdi et al., 2022; Zeng et al., 2022; Zhu et al., 2023). Previous studies have certificated that miRNAs also play an essential part in BAT as transcriptional regulators and biomarkers (Goody and Pfeifer, 2019). For instance, miR-34C-5P was proven to regulate DPYSL4 expression and have an influence on insulin β-cells, the inflammatory response and glucose oxidative catabolism (Qiu et al., 2023). Elevated levels of miR-210, induced by both hypoxia and adrenergic stimulation, played a key role in regulating the differentiation and thermogenic function of brown adipocytes (Caca et al., 2025). Nevertheless, no studies have investigated miRNA networks in OSA-mediated BAT dysfunction.

This study pioneers the profiling of miRNA alterations in BAT during OSA progression. Using an ApoE mouse model of CIH, we integrated miRNA sequencing, bioinformatics, and experimental validation to identify dysregulated miRNAs and their mechanistic roles in BAT dysfunction. Finally, a DemiRs and target mRNA regulatory network was construed. Our study may pave a novel strategy for understanding molecular mechanisms of OSA-associated MS.

## Materials and methods

### Animals

APOE-deficient mice were chosen for their heightened susceptibility to metabolic dysfunction, including insulin resistance and dyslipidemia, which mirrors OSA-associated metabolic syndrome in humans. Male APOE mice were obtained from Guangdong Yaokang Biological Technology Co., Ltd. Approval was granted to all experiments by the Institutional Animal Care and Use Committee of the Second Affiliated Hospital of Fujian Medical University.

### Chronic intermittent hypoxia system

The CIH protocol was adapted from established models of OSA (Chen et al., 2025; Zhang et al., 2025). APOE male mice were assigned to CIH or normal in random and exposed for 8 weeks in an environmental chamber applying a gas control system. To replicate CIH that experienced by patients with OSA, for 8 weeks, mice in the CIH group were kept in a device that produced 60 hypoxic episodes each hour (40 s of room air exposure followed by 20 s of 5% oxygen) for 8 hours each day. The normal group was placed in the same apparatus, and regular air was supplied to the apparatus.

### miRNAs-seq and bioinformatics analysis

To obtain miRNAs and mRNA expression profiles, total RNA extraction from BAT was carried out. BAT samples were homogenized in 1 mL TRI Reagent. Phase separation was achieved by adding 200 μL chloroform, followed by centrifugation for 15 min at 4°C. The aqueous phase was transferred, and RNA was precipitated with 500 μL isopropanol, washed twice with 75% ethanol, and dissolved in RNase-free water. RNA concentration and purity were verified via NanoDrop. RNA integrity was assessed using Agilent Bioanalyzer 2100, and only samples with an RNA Integrity Number (RIN) >7.0 were used for sequencing. Total RNA (1 μg) was reverse-transcribed into cDNA using the PrimeScript™ RT Reagent Kit with gDNA Eraser, following the manufacturer’s protocol. Reactions included: 37°C for 15 min, 85°C for 5 s, and 4°C hold. The small RNA library was established with the aid of the NEB Multiplex Small RNA Library Prep Set for Illumina, according to the manufacturer’s instructions. Differentially expressed genes and DEmiRs were detected by software edgeR (v3.40.2) with thresholds: |log_2_FC| > 1.0 and adjusted p value <0.05,and then were visualized on a volcano plot. Gene Ontology (GO) and Kyoto Encyclopedia of Genes and Genomes (KEGG) pathway enrichment analyses on the parental genes of the miRNAs were conducted to analyze the functions of the DEmiRs. Targeted mRNAs of the DEmiRs were analyzed using the GO and KEGG databases to show the vital functions and pathways.

### Real-time quantitative PCR assay (RT-qPCR)

The extraction of all RNA from BAT was performed using TRI Reagent (Sigma: T9424) in accordance with the manufacturer’s protocol and reverse transcription was undertaken using a QuantStudio5 Real-time PCR System. RT-qPCR was performed using 2×PCR master mix (Arraystar: AS-MR-006-5) on a QuantStudio5 System (Applied Biosystems). Reaction conditions: 95°C for 10 min; 40 cycles of 95°C for 10 s and 60°C for 60 s. Each reaction contained 5 μL 2×Master Mix, 1.0 μL primers (10 μM), 2 μL cDNA, and 2.0 μL nuclease-free water. For RT-qPCR, data were normalized to the endogenous control U6 snRNA using the 2^−ΔΔCT^ method. Each experiment was carried out three times. The primer sequences can be seen in Table 1.

**TABLE 1:** RT-qPCR primers.

Gene	Sequence (5'->3′)	Length (bp)
U6	F:5′GCTTCGGCAGCACATATACTAAAAT3′ R:5′CGCTTCACGAATTTGCGTGTCAT3′	89
mmu-miR-3102-3p	GSP:5′GCAGAGCACCCCATTGGC3′ R:5′GTGCGTGTCGTGGAGTCG3′	64
mmu-miR-135a-5p	GSP:5′GGGGTATGGCTTTTTATTCCT3′ R:5′GTGCGTGTCGTGGAGTCG3′	65
mmu-miR-92b-3p	GSP:5′GGTATTGCACTCGTCCCG3′ R:5′GTGCGTGTCGTGGAGTCG3′	62
mmu-miR-185-3p	GSP:5′GGTTAGGGGCTGGCTTTC3′ R:5′GTGCGTGTCGTGGAGTCG3′	64
mmu-miR-505-3p	GSP:5′GGTCGTCAACACTTGCTGG3′ R:5′GTGCGTGTCGTGGAGTCG3′	63
mmu-miR-222-5p	GSP:5′GGGGTCTCAGTAGCCAGTGT3′ R:5′GTGCGTGTCGTGGAGTCG3′	64

### miRNAs targets prediction

Remarkably enriched KEGG pathways (P < 0.05) were identified for the predicted target genes using the Kyoto Encyclopedia of Genes and Genome. Besides, combining the predicted target genes with the use of miRDB (v6.0) and Targeted Scan (v7.0) and the DEmiRs for these miRNAs, a core miRNA target network was built by using the Cytoscape software (v3.9.1).

### Statistical analysis

Every figure was expressed as the mean ± standard deviation. Unpaired Student’s t-tests were used to compare CIH groups with normal groups. In order to qualify as a notable difference, the P value had to be less than 0.05. Meanwhile, we used FDR (False Discovery Rate) correction via the Benjamini–Hochberg method to account for multiple testing. Differential expression was defined as adjusted p-value <0.05 and |log_2_FC| > 1.0.

## Results

### Differentially expressed miRNAs

**FIGURE 1:**
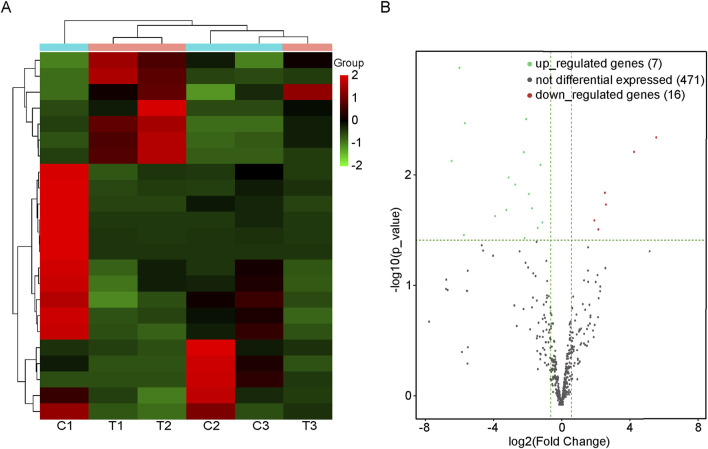
DEmiRs in BAT of mice with or without OSA. **(A)** Heat map of the DEmiRs in mice with or without OSA. C1, C2, and C3 were control groups; T1, T2, and T3 were OSA groups. **(B)** The volcano plot reveals the DEmiRs between the OSA mice and the control mice. Those dots that are red represent miRNAs that are upregulated, while those that are green represent miRNAs that are downregulated.

A total of 23 DEmiRs were found in the BAT of the CIH group compared with the normal group. In CIH-induced BAT injury models, 7 miRNAs were significantly upregulated (e.g., mmu-miR-3102-3p: log_2_FC = 4.171, adjusted p value = 0.010), and 16 miRNAs were downregulated (e.g., mmu-miR-185-3p: log_2_FC = −3.145, adjusted p value = 0.029). A comprehensive list of all differentially expressed miRNAs was provided in Supplementary Table S1. Results of cluster analysis and volcano plot analysis of all DEmiRs were displayed respectively in Figures 1A,B. The gene expression variation between the CIH and control group is presented in Supplementary Figure S1A. The chromosomal distribution of these differentially expressed genes (DEGs) is shown in Supplementary Figure S1B.

### Enrichment analysis for the target genes of demiRs

**FIGURE 2:**
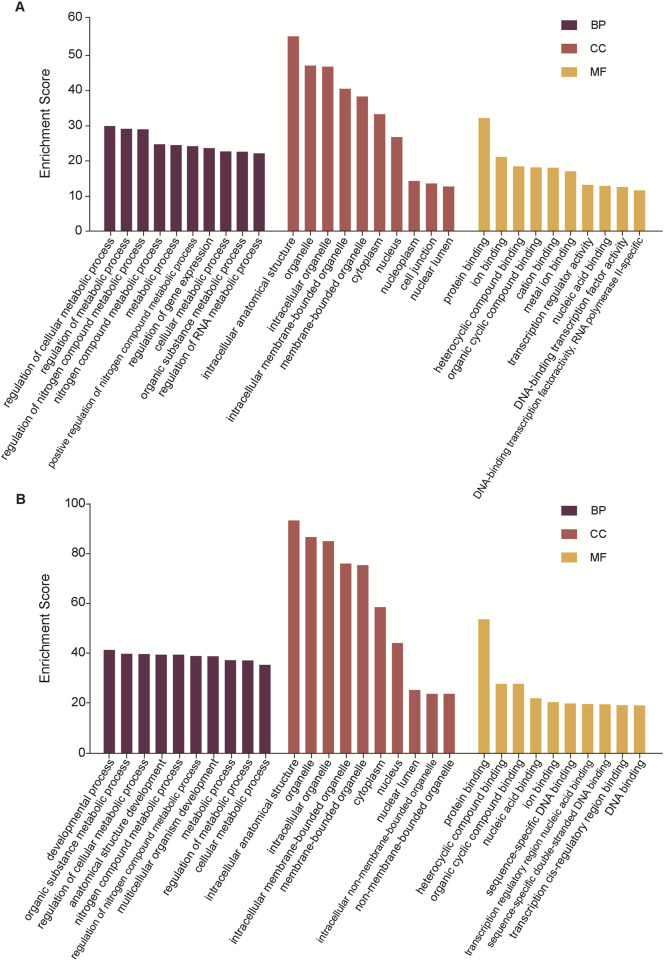
GO analysis. **(A)** The GO terms for the DemiRs that are upregulated. **(B)** The GO terms for the DemiRs that are downregulated.

**FIGURE 3:**
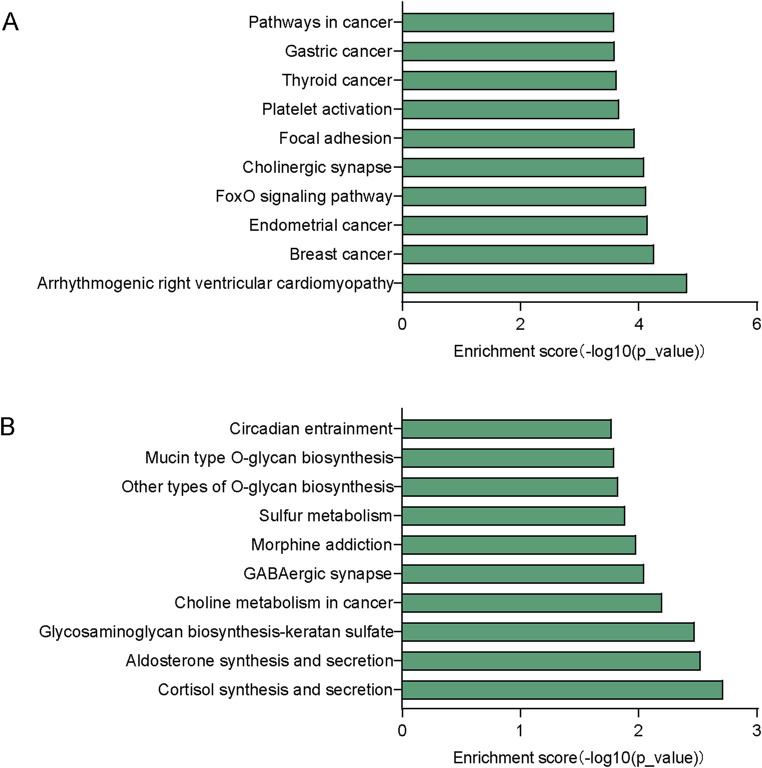
KEGG pathway analysis. **(A)** Top 10 significant pathways associated with upregulated genes. **(B)** Top 10 significant pathways related to downregulated genes.

To gain more information about the potential mechanisms of DEmiRs, GO and KEGG pathway enrichment analyses were conducted on target genes of all 23 DEmiRs. Results of the GO analysis of DEmiRs target genes are displayed in Figure 2. In order to analyze predicted genes, the corresponding GO annotations were examined, which include information about cellular components (CC), biological processes (BP), and molecular functions (MF). The analysis demonstrated that these genes were implicated in metabolic process, such as regulation of cellular metabolic process, regulation of nitrogen compound metabolic process, organic substance metabolic process, and regulation of RNA metabolic process. KEGG Pathway analysis was used to identify enriched pathways based on representative profiles of miRNA targeted genes. The analysis revealed that some of these genes were involved in signal transduction pathways (Figure 3), including focal adhesion kinase, FoxO signaling pathway, Sulfur metabolism, Cholinergic synapse and Choline metabolism in cancer.

### RT-qPCR validation of DEmiRs

**FIGURE 4:**
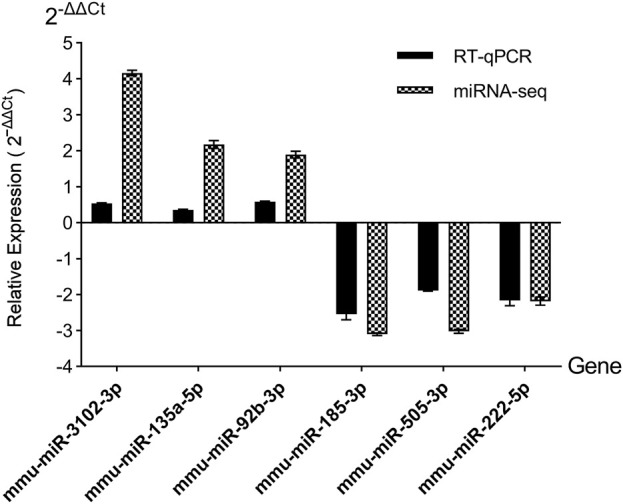
RT-qPCR validation of candidate DEmiRs. miRNAs-seq, miRNAs sequencing. Relative expression was calculated using the 2^−ΔΔCT^ method, normalized to U6 snRNA.

Then the accurateness and reliability of the sequencing analysis were validated using RT-qPCR. Based on their magnitude of expression change, established roles in metabolic pathways, and practical constraints, we chose three upregulated miRNAs (mmu-miR-3102-3p, mmu-miR-135a-5p, mmu-miR-92b-3p) and three downregulated miRNAs (mmu-miR-185-3p, mmu-miR-505-3p, mmu-miR-222-5p). As shown in Figure 4, expression profiles of these selected miRNAs show a similar trend with the results from our RNA-Seq analysis, which means the reliability of our miRNAs-seq data was high.

### miRNA/mRNA interaction network analysis

**FIGURE 5:**
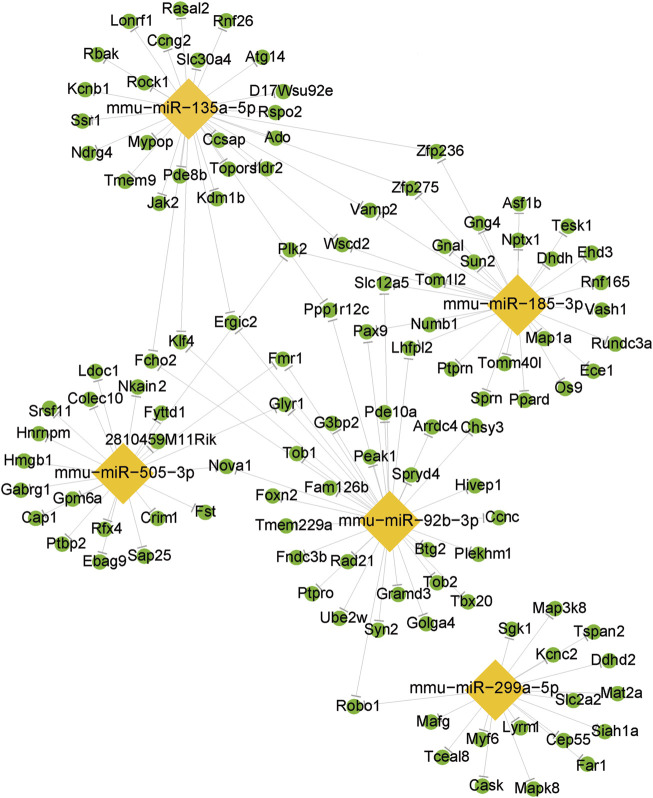
The differentially expressed miRNA/mRNA interaction network. The lozenge represents the DEmiRs. The circle represents the target mRNAs.

Based on RT-qPCR validation and relevance to OSA-associated metabolic syndrome, five miRNAs were selected for miRNA-mRNA network analysis (Figure 5). The integrated miRNA-mRNA network was built based on the dependence of these miRNAs on their targeted genes. This network provides a comprehensive understanding of the interaction between miRNAs and their targeted genes. By revealing their targeted mRNAs, we can investigate the fundamental mechanism of these DemiRs.

## Discussion

More and more studies indicate that there exist a complicated and bidirectional association between OSA and MS (Framnes and Arble, 2018; Karuga et al., 2023; Martins and Conde, 2021). OSA leads to and exacerbates metabolic disorders, and cardiovascular dysfunction. CIH, the hallmark feature of OSA, triggers cyclic oxygen deprivation/reoxygenation, directly inducing BAT dysfunction through three key mechanisms: insulin resistance, sympathetic activation, or inflammation. It was reported that CIH-driven hepatic inflammation, mitochondrial dysfunction, and oxidative stress critically contributed to the development of MS associated with OSA (Fernandes et al., 2023). MS is also considered an emerging major risk factor for the development of OSA. According to a report, the incidence of OSA is high among those with MS, as well as MS prevalence among those with OSA, which ranges from 60% to 70% (Giampá et al., 2023). Short-chain fatty acids from gut microbiota might influence MS in OSA via immune system regulation, as suggested by studies linking gut microbiota to OSA-associated hypertension (Zhang et al., 2022). This feature can trigger intestinal oxidative stress and inflammation, ultimately culminating in OSA-related MS (Shobatake et al., 2022). However, the definite underlying mechanisms of OSA-related MS remain incompletely understood.

The discovery of miRNAs in diseases has opened new vistas for researchers in recent years. In addition to post-transcriptional regulation of gene expression, miRNAs have a range of physiological effects (Zhang et al., 2020). Furthermore, miRNAs are being identified as crucial regulators of numerous pathological and physiological processes and have been reported to have an impact on various diseases. However, the precise contribution of miRNAs to the pathogenesis of OSA-related MS remains unclear. Thus, we constructed CIH-induced BAT injury models to uncover the underlying mechanisms of miRNAs in OSA-related MS.

The aim of this study was to analyze the abnormal miRNAs profiles using RNA-seq in the CIH mouse models. The findings of this study revealed that a total of 23 DEmiRs were identified and selected. Of them, 7 miRNAs were upregulated. In addition to the upregulated miRNAs, we obtained 16 downregulated miRNAs. Notably, downregulated miR-505-3p, previously linked to macrophage dysfunction in hypercholesterolemia (Escate et al., 2018), may exacerbate inflammation in OSA-related BAT injury. Conversely, upregulated miR-135a-5p, which targets insulin signaling pathways (Teichenne et al., 2015), could drive metabolic dysregulation. These findings extend prior work on OSA-gut microbiota-metabolism interactions (Xue et al., 2021) by implicating BAT-specific miRNA networks. The RT-qPCR results were in accord with the miRNAs sequencing data. We also discovered that a diverse range of biological and pathological processes have also been linked to these identified miRNAs in this study. For example, the presence of miR-16-2-3p in diabetes is linked to coronary microvascular dysfunction by controlling the breakdown of fatty acids in endothelial cells (Liu et al., 2024). Nevertheless, further investigation of these DEmiRs is needed to understand the role they play in the progression of CIH-related MS.

It was necessary to identify potential target genes for these DEmiRs and to determine likely signaling pathways. Our research revealed that these DEmiRs may potentially regulate 107 pathways. For the upregulated DEmiRs, the arrhythmogenic right ventricular cardiomyopathy pathway was the most enriched one, which indicates that OSA has a great impact on cardiovascular disease (CVD). Recent reports have stated that MS is a clustering of metabolic complications that increase the risk of CVD (Kuckuck et al., 2024). Among the downregulated DemiRs cortisol synthesis and secretion pathway was the most enriched. Previous studies showed that repeated physiological stress such as hypoxia and fragmentation of sleep can affect cortisol secretion (Mohammadi et al., 2020). Meanwhile, the development of MS may be influenced by stress and its related hormones, such as cortisol, the glucocorticoid hormone (Kuckuck et al., 2024). However, the precise underlying mechanism between OSA and MS remains uncertain.

Then we identified the crucial miRNAs and mRNAs based on the quantity of their neighbors or connections. By conducting an intricate investigation of the miRNA-mRNA network, we found that a single miRNA exerted control over a sequence of target genes, while many miRNAs regulated several target genes. Prior to studying miRNA-regulated genes, we should also pay attention to mRNAs and genes regulated by several miRNAs. On the other hand, miRNAs regulating a larger number of mRNAs/genes have higher possibilities to influence a pathway and perform their biological functions. While the diverse nature of miRNA-mRNA interactions is well-documented, our network analysis identified miR-135a-5p as a high-connectivity hub (regulating three targets in arrhythmogenic pathways, Figure 4). This positions it as a candidate for future mechanistic studies of OSA-related cardiovascular complications, such as via *in vivo* knockout models. We emphasize that these findings are exploratory and require functional validation to validate the specific roles of the miRNA-mRNA network in OSA-related MS, further research is needed.

There are multiple constraints that need to be acknowledged in this study. First, there was no investigation of the function and relevance of identified DemiRs. Second, the study did not include female mice, hence the potential impact of gender could not be ruled out. Third, while validating all 23 DEmiRs experimentally is ideal, this was not feasible due to sample limitations. Future studies will expand validation. Fourth, our study relies exclusively on CIH to model OSA. While CIH effectively induces hypoxia-reoxygenation cycles analogous to OSA, it does not replicate sleep fragmentation or neurocognitive arousals-critical elements of clinical OSA pathophysiology. Recent studies demonstrate that combined models (e.g., CIH + sleep disruption) better mimic multisystemic OSA manifestations. For instance, Yin et al. (Wang et al., 2022) showed that adding sleep fragmentation to CIH exacerbates metabolic dysfunction beyond hypoxia alone. This may limit direct translation to human OSA complexity. Fifth, the RT-qPCR validation is limited to 6 miRNAs. Luciferase reporter assays and functional studies *in vitro*/*in vivo* will be essential to confirm causal relationships between key DEmiRs and BAT dysfunction. Last but not least, the limited number of samples makes the generalized results difficult to achieve.

## Conclusion

To summarize, an abundance of DEmiRs was found in BAT of mouse models with CIH and a functional interaction network was identified between them. These findings broadened our present understanding of the molecular etiology of OSA-related MS, which could make a contribution to the development of novel therapeutic strategies for this disease.

## Data Availability

The original contributions presented in the study are included in the article/Supplementary Material, further inquiries can be directed to the corresponding authors.

## Supplementary material

SUPPLEMENTARY FIGURE S1
**(A)** DEGs are presented on a scatter plot. Respectively, Green and red dots indicated decreased and increased expressed genes in BAT. **(B)** The distribution plot indicates the distribution of DEGs in chromosomes. Green and red indicated downregulated and upregulated genes in the BAT of the CIH mouse model, respectively.

## References

[B1] Almendros I. Basoglu Ö. K. Conde S. V. Liguori C. Saaresranta T. (2022). Metabolic dysfunction in osa: is there something new under the sun? J. Sleep. Res. 31 (1), e13418. 10.1111/jsr.13418 34152053

[B2] Caca J. Bartelt A. Egea V. (2025). Hypoxia regulates brown adipocyte differentiation and stimulates mir-210 by hif-1α. Int. J. Mol. Sci. 26 (1), 117. 10.3390/ijms26010117 PMC11720532 39795975

[B3] Chen Q. Lai H. Chen Y. Peng Z. Wu S. Liu D. (2025). Characterization of circrna expression profiles and functional roles in a mouse model of liver injury induced by osa. Sci. Rep.-UK 15 (1), 15615. 10.1038/s41598-025-99612-6 PMC12050294 40320447

[B4] Cui F. Guan Y. Guo J. Tian Y. Hu H. Zhang X. (2018). Chronic intermittent hypobaric hypoxia protects vascular endothelium by ameliorating autophagy in metabolic syndrome rats. Life Sci. 205, 145–154. 10.1016/j.lfs.2018.05.008 29733850

[B5] Dahan T. Nassar S. Yajuk O. Steinberg E. Benny O. Abudi N. (2022). Chronic intermittent hypoxia during sleep causes browning of interscapular adipose tissue accompanied by local insulin resistance in mice. Int. J. Mol. Sci. 23 (24), 15462. 10.3390/ijms232415462 36555109 PMC9779339

[B6] Dong M. Lin J. Lim W. Jin W. Lee H. J. (2018). Role of brown adipose tissue in metabolic syndrome, aging, and cancer cachexia. Front. Med. 12 (2), 130–138. 10.1007/s11684-017-0555-2 29119382

[B7] Escate R. Mata P. Cepeda J. M. Padreó T. Badimon L. (2018). Mir‐505‐3p controls chemokine receptor up‐regulation in macrophages: role in familial hypercholesterolemia. FASEB J. 32 (2), 601–612. 10.1096/fj.201700476R 32172543

[B8] Fernandes J. L. Martins F. O. Olea E. Prieto-Lloret J. Braga P. C. Sacramento J. F. (2023). Chronic intermittent hypoxia-induced dysmetabolism is associated with hepatic oxidative stress, mitochondrial dysfunction and inflammation. Antioxidants (Basel) 12 (11), 1910. 10.3390/antiox12111910 38001763 PMC10669005

[B9] Framnes S. N. Arble D. M. (2018). The bidirectional relationship between obstructive sleep apnea and metabolic disease. Front. Endocrinol. (Lausanne) 9, 440. 10.3389/fendo.2018.00440 30127766 PMC6087747

[B10] Giampá S. Q. C. Lorenzi Filho G. Drager L. F. (2023). Obstructive sleep apnea and metabolic syndrome. Obesity 31 (4), 900–911. 10.1002/oby.23679 36863747

[B11] Goody D. Pfeifer A. (2019). Micrornas in brown and beige fat. Biochimica Biophysica Acta (BBA) - Mol. Cell Biol. Lipids 1864 (1), 29–36. 10.1016/j.bbalip.2018.05.003 29758288

[B12] Huang X. Huang X. Guo H. Li J. Zhou C. Huang Y. (2022). Intermittent hypoxia-induced mettl3 downregulation facilitates mgll-mediated lipolysis of adipocytes in osas. Cell Death Discov. 8 (1), 352. 10.1038/s41420-022-01149-4 35933406 PMC9357002

[B13] Karuga F. F. Jaromirska J. Malicki M. Sochal M. Szmyd B. Białasiewicz P. (2023). The role of micrornas in pathophysiology and diagnostics of metabolic complications in obstructive sleep apnea patients. Front. Mol. Neurosci. 16, 1208886. 10.3389/fnmol.2023.1208886 37547923 PMC10403239

[B14] Kuckuck S. Lengton R. Boon M. R. Boersma E. Penninx B. W. J. H. Kavousi M. (2024). Long‐term glucocorticoids in relation to the metabolic syndrome and cardiovascular disease: a systematic review and meta‐analysis. J. Intern. Med. 295 (1), 2–19. 10.1111/joim.13739 37926862

[B15] Liu Y. Zhong C. Chen S. Xue Y. Wei Z. Dong L. (2024). Circulating exosomal mir-16-2-3p is associated with coronary microvascular dysfunction in diabetes through regulating the fatty acid degradation of endothelial cells. Cardiovasc. Diabetol. 23 (1), 60. 10.1186/s12933-024-02142-0 38336726 PMC10858495

[B16] Martins F. O. Conde S. V. (2021). Gender differences in the context of obstructive sleep apnea and metabolic diseases. Front. physiology 12, 792633. 10.3389/fphys.2021.792633 PMC8712658 34970158

[B17] Mohammadi H. Rezaei M. Sharafkhaneh A. Khazaie H. Ghadami M. R. (2020). Serum testosterone/cortisol ratio in people with obstructive sleep apnea. J. Clin. Lab. Anal. 34 (1), e23011. 10.1002/jcla.23011 31549459 PMC6977109

[B18] Naqvi R. A. Datta M. Khan S. H. Naqvi A. R. (2022). Regulatory roles of microrna in shaping t cell function, differentiation and polarization. Semin. Cell Dev. Biol. 124, 34–47. 10.1016/j.semcdb.2021.08.003 34446356 PMC11661912

[B19] Qiu L. Sheng P. Wang X. (2023). Identification of metabolic syndrome-related mirna–mrna regulatory networks and key genes based on bioinformatics analysis. Biochem. Genet. 61 (1), 428–447. 10.1007/s10528-022-10257-w 35877019

[B20] Saito M. Okamatsu-Ogura Y. (2023). Thermogenic brown fat in humans: implications in energy homeostasis, obesity and metabolic disorders. World J. Mens. Health 41 (3), 489–507. 10.5534/wjmh.220224 36792089 PMC10307652

[B21] Sapiña-Beltrán E. Gracia-Lavedan E. Torres G. Gaeta A. M. Paredes J. Mayoral A. (2022). Prevalence of obstructive sleep apnoea and its association with atherosclerotic plaques in a cohort of subjects with mild–moderate cardiovascular risk. Arch. Bronconeumología 58 (6), 490–497. 10.1016/j.arbres.2021.01.026 33741145

[B22] Sharma S. Stansbury R. Hackett B. Fox H. (2021). Sleep apnea and pulmonary hypertension: a riddle waiting to be solved. Pharmacol. Ther. 227, 107935. 10.1016/j.pharmthera.2021.107935 34171327

[B23] Shobatake R. Ota H. Takahashi N. Ueno S. Sugie K. Takasawa S. (2022). The impact of intermittent hypoxia on metabolism and cognition. Int. J. Mol. Sci. 23 (21), 12957. 10.3390/ijms232112957 36361741 PMC9654766

[B24] Tan X. Zhu T. Zhang L. Fu L. Hu Y. Li H. (2022). Mir-669a-5p promotes adipogenic differentiation and induces browning in preadipocytes. Adipocyte 11 (1), 120–132. 10.1080/21623945.2022.2030570 35094659 PMC8803067

[B25] Tealdi S. Ferro E. Campa C. C. Bosia C. (2022). Microrna-mediated encoding and decoding of time-dependent signals in tumorigenesis. Biomolecules 12 (2), 213. 10.3390/biom12020213 35204714 PMC8961662

[B26] Teichenne J. Morro M. Casellas A. Jimenez V. Tellez N. Leger A. (2015). Identification of mirnas involved in reprogramming acinar cells into insulin producing cells. PLoS One 10 (12), e0145116. 10.1371/journal.pone.0145116 26690959 PMC4686894

[B27] Wang F. Zou J. Xu H. Huang W. Zhang X. Wei Z. (2022). Effects of chronic intermittent hypoxia and chronic sleep fragmentation on gut microbiome, serum metabolome, liver and adipose tissue morphology. Front. Endocrinol. (Lausanne) 13, 820939. 10.3389/fendo.2022.820939 35178032 PMC8846366

[B28] Xue J. Allaband C. Zhou D. Poulsen O. Martino C. Jiang L. (2021). Influence of intermittent hypoxia/hypercapnia on atherosclerosis, gut microbiome, and metabolome. Front. physiology 12, 663950. 10.3389/fphys.2021.663950 PMC8060652 33897472

[B29] Zeng H. Huang M. Gong X. (2022). Microrna-124-3p promotes apoptosis and autophagy of glioma cells by down-regulating crebrf. Neurol. Res. 44 (12), 1094–1103. 10.1080/01616412.2022.2112374 35981103

[B30] Zhang X. Lin X. Wu X. Zeng Y. Chen X. Luo X. (2020). Differential expression of micrornas in xenografted lewis lung carcinomas subjected to intermittent hypoxia: a next-generation sequence analysis. Transl. cancer Res. 9 (7), 4354–4365. 10.21037/tcr-19-2913 35117801 PMC8798178

[B31] Zhang L. Ko C. Zeng Y. (2022). Immunoregulatory effect of short-chain fatty acids from gut microbiota on obstructive sleep apnea-associated hypertension. Nat. Sci. sleep 14, 393–405. 10.2147/NSS.S354742 35299627 PMC8922759

[B32] Zhang J. Guo Y. Ji M. Lin S. Liu D. Chen Q. (2025). A comprehensive analysis of microrna alteration in an apoe(−/−) mice model of white adipose tissue injury induced by chronic intermittent hypoxia. Front. Genet. 16, 1474223. 10.3389/fgene.2025.1474223 40206502 PMC11979184

[B33] Zhu J. Xu Z. Wu P. Zeng C. Peng C. Zhou Y. (2023). Microrna-92a-3p inhibits cell proliferation and invasion by regulating the transcription factor 21/steroidogenic factor 1 axis in endometriosis. Reprod. Sci. 30 (7), 2188–2197. 10.1007/s43032-021-00734-9 36650372 PMC10310800

