# Supplementary material for: Differential expression profiles and bioinformatics analysis of microRNAs in brown adipose tissue dysfunction induced by chronic intermittent hypoxia in obstructive sleep apnea
Source: Front Cell Dev Biol. 2025 Aug 15;13:1598018. doi: 10.3389/fcell.2025.1598018 (PMC12395453; doi:10.3389/fcell.2025.1598018)
Supplement: Supplementary file 1 [file Table1.docx]

TABLE 2 All differentially expressed miRNAs.

| Gene | log_2_ Fold Change | adjusted p value | Regulation |
| --- | --- | --- | --- |
| mmu-miR-1929-5p | 5.439 | 0.007 | Up |
| mmu-miR-3102-3p | 4.171 | 0.010 | Up |
| mmu-miR-1960 | 2.942 | 0.006 | Up |
| mmu-miR-5100 | 2.563 | 0.027 | Up |
| mmu-miR-16-2-3p | 2.497 | 0.021 | Up |
| mmu-miR-135a-5p | 2.125 | 0.042 | Up |
| mmu-miR-92b-3p | 1.895 | 0.035 | Up |
| mmu-miR-7214-5p | -6.269 | 0.012 | Down |
| mmu-miR-466m-3p | -5.821 | 0.002 | Down |
| mmu-miR-871-5p | -5.574 | 0.046 | Down |
| mmu-miR-871-3p | -5.519 | 0.006 | Down |
| mmu-miR-7092-5p | -3.779 | 0.032 | Down |
| mmu-miR-185-3p | -3.145 | 0.029 | Down |
| mmu-miR-505-3p | -3.009 | 0.016 | Down |
| mmu-miR-34b-3p | -2.635 | 0.018 | Down |
| mmu-miR-222-5p | -2.144 | 0.010 | Down |
| mmu-miR-299a-5p | -2.099 | 0.049 | Down |
| mmu-miR-376b-3p | -2.004 | 0.006 | Down |
| mmu-miR-3963 | -1.850 | 0.023 | Down |
| mmu-miR-3470a | -1.669 | 0.027 | Down |
| mmu-miR-19a-3p | -1.348 | 0.040 | Down |
| mmu-miR-30e-5p | -1.179 | 0.013 | Down |
| mmu-miR-19b-3p | -1.079 | 0.037 | Down |
