# Supplementary figures and images for: Differential expression profiles and bioinformatics analysis of microRNAs in brown adipose tissue dysfunction induced by chronic intermittent hypoxia in obstructive sleep apnea
Source: Front Cell Dev Biol. 2025 Aug 15;13:1598018. doi: 10.3389/fcell.2025.1598018 (PMC12395453; doi:10.3389/fcell.2025.1598018)

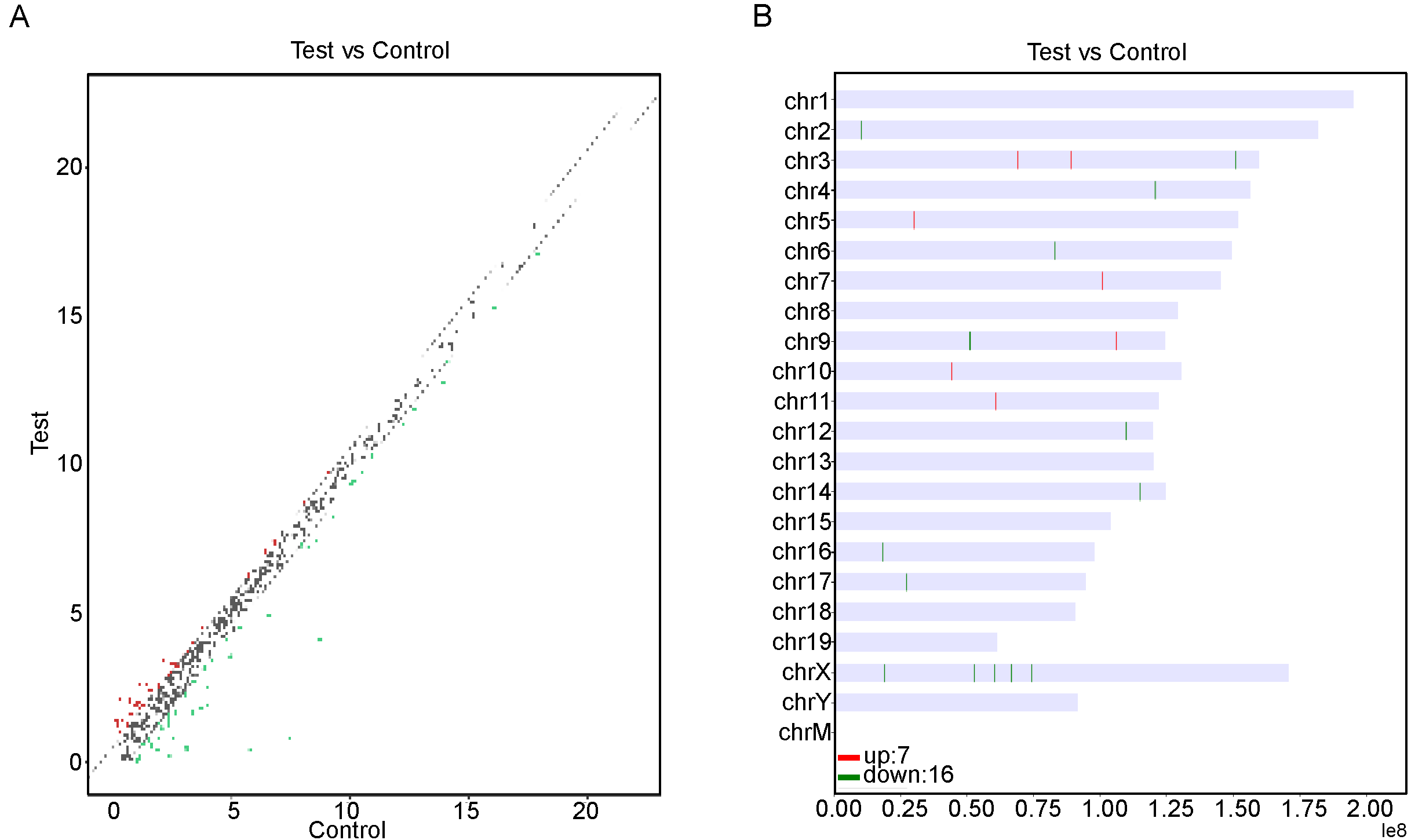

Supplement: Supplementary file 2 [file Image1.tif]
